# Linoleic acid participates in the response to ischemic brain injury through oxidized metabolites that regulate neurotransmission

**DOI:** 10.1038/s41598-017-02914-7

**Published:** 2017-06-28

**Authors:** Marie Hennebelle, Zhichao Zhang, Adam H. Metherel, Alex P. Kitson, Yurika Otoki, Christine E. Richardson, Jun Yang, Kin Sing Stephen Lee, Bruce D. Hammock, Liang Zhang, Richard P. Bazinet, Ameer Y. Taha

**Affiliations:** 1 Department of Food Science and Technology, College of Agriculture and Environmental Sciences, University of California, Davis, CA USA; 2 Department of Nutritional Sciences, Faculty of Medicine, University of Toronto, ON, Canada; 3 Food and Biodynamic Laboratory, Graduate School of Agricultural Science, Tohoku University, Sendai, Miyagi Japan; 4 Department of Nutrition, College of Agriculture and Environmental Sciences, University of California, Davis, CA USA; 5 Department of Entomology and Nematology, College of Agriculture and Environmental Sciences and Comprehensive Cancer Center, Medical Center, University of California, Davis, CA USA; 6 Krembil Research Institute, University Health Network, Toronto, ON Canada; 7 Department of Medicine (Neurology), University of Toronto, ON, Canada

## Abstract

Linoleic acid (LA; 18:2 n-6), the most abundant polyunsaturated fatty acid in the US diet, is a precursor to oxidized metabolites that have unknown roles in the brain. Here, we show that oxidized LA-derived metabolites accumulate in several rat brain regions during CO_2_-induced ischemia and that LA-derived 13-hydroxyoctadecadienoic acid, but not LA, increase somatic paired-pulse facilitation in rat hippocampus by 80%, suggesting bioactivity. This study provides new evidence that LA participates in the response to ischemia-induced brain injury through oxidized metabolites that regulate neurotransmission. Targeting this pathway may be therapeutically relevant for ischemia-related conditions such as stroke.

## Introduction

Omega-6 linoleic acid (LA, 18:2 n-6) is the most consumed polyunsaturated fatty acid (PUFA) in the US diet, accounting for approximately 7% of daily calories^1^. The consumption of its elongation-desaturation product, arachidonic acid (AA, 20:4n-6), as well as omega-3 α-linolenic acid (ALA, 18:3n-3), eicosapentaenoic acid (EPA, 20:5n-3) and docosahexaenoic acid (DHA, 22:6n-3), collectively account for less than 1% of calories. Despite being the main PUFA in the diet, little is known about the role of LA in the brain.

Most brain studies have focused on AA and DHA, because they are enriched in phospholipid membranes, and are known to regulate many processes including blood flow^2–4^, pain signaling^5–7^, inflammation^8^ and the resolution of inflammation^9–13^ through oxidized metabolites known as oxylipins. PUFA-derived oxylipins are synthesized via lipoxygenase (LOX)^14–16^, cyclooxygenase (COX)^15, 17, 18^, cytochrome P450 (CYP450)^19–21^ or soluble epoxide hydrolase (sEH) enzymes^6, 22^ following phospholipase-mediated release of fatty acids from brain membrane phospholipids^23, 24^. Oxylipin synthesis can also occur non-enzymatically^25–27^.

Unlike AA and DHA, which make up over 20% of brain total fatty acids, LA accounts for less than 2% of total fatty acids^28^, but enters the brain at a comparable rate to AA and DHA (4–7 pmol/g/s)^29, 30^. Instead of incorporating into membrane phospholipids, however, up to 59% of the LA entering the brain is converted into relatively polar compounds^29^, which include LA-derived oxylipins^31^ produced non-enzymatically or via the same LOX, COX, CYP450 and sEH enzymes that act on AA and DHA^17, 32–34^.

Brain injury caused by hypoxia, ischemia, seizures or trauma activates excitatory N-methyl-D-aspartate (NMDA) receptors coupled to phospholipase enzymes^35–37^, which release AA and DHA from membrane phospholipids^38–43^. The majority of unesterified AA and DHA are re-esterified into the phospholipid membrane^44^, whereas a small portion (~3%) is converted via non-enzymatic or enzymatic pathways into oxylipins^41, 45–47^ that regulate the brain’s response to injury^2–4^. This response involves oxylipins that acutely down-regulate neuronal hyperexcitability^48^ and enhance vasodilation^49^ as a protective mechanism.

Brain unesterified LA concentration also increases following brain injury^24, 40^, suggesting that LA or its metabolites may be involved in the response to brain injury. However, very little is known about the role of LA or its metabolites in brain. LA was reported to raise seizure threshold in rats^50, 51^, and to increase the number and duration of spontaneous wave discharges in a rat model of absence seizures^51^, suggesting its involvement in neurotransmission. Although it is not known whether the effects of LA in brain are mediated by LA itself or its oxidized metabolites, LA-metabolites have been detected in brain tissue^31, 52^ and are known to activate pain-gating transient receptor potential vanilloid (TRPV) channels and inflammatory pathways in rodent spinal cord^53^ and hindpaw^54^, and to reduce retinal epithelial cell growth^55^. These studies suggest that LA metabolites are likely bioactive in brain. Understanding the conditions that increase the formation of LA-derived metabolites and whether they are bioactive in brain may inform on new pathways that could be targeted.

The present study tested the hypothesis that LA partakes in the response to ischemic brain injury through oxidized metabolites that regulate brain signaling. A targeted lipidomics approach involving liquid chromatography tandem mass-spectrometry (LC-MS/MS) was used to quantify 85 PUFA-derived oxylipins (listed in Supplementary Table 1) in cortex, hippocampus, cerebellum and brainstem of rats subjected to CO_2_ asphyxiation-induced ischemia or head-focused microwave (MW) fixation, which heat-denatures enzymes to halt brain lipid metabolism^56, 57^. These brain regions were chosen because they are particularly affected to varying degrees by hypoxic or ischemic insults^58–65^. The lipidomic method used herein, extensively covered LA, AA and DHA metabolites, to contrast the ischemia-induced response of LA metabolites to published data on the AA and DHA metabolites produced during ischemic injury. It also included metabolites derived from other minor fatty acids in brain, such as ALA, EPA and di-homo-gamma-linolenic acid (DGLA), an intermediate elongation-desaturation product of LA, because we intended to assess whether they also participate in the response to ischemic brain injury.

The effects of AA, AA-derived prostaglandin E_2_ (PGE_2_), LA and LA-derived 13-hydroxyoctadecadienoic acid (13-HODE) on hippocampal paired-pulse facilitation (PPF), a marker of short-term plasticity^66^, were measured using extracellular recordings to test whether 13-HODE regulates neurotransmission in a manner comparable to PGE_2_, a well-studied lipid mediator involved in hippocampal signaling^67–69^. 13-HODE was tested upon finding that its concentration increased in cortex and brainstem following ischemia, and that it is the most abundant LA-metabolite detected in rat hippocampus. Extracellular recordings were measured from hippocampus because of its clearly-defined structural attributes and robust signals which enable accurate extracellular recordings and assessment of changes in neurotransmission. It is also vulnerable to the neurodegenerative effects of ischemic injury^58–61^.

## Results

### Ischemia induced global changes in oxylipin concentrations

Targeted LC-MS/MS analysis detected the presence of 53, 34, 43 and 37 oxylipins in cortex, hippocampus, cerebellum and brainstem, respectively. As shown in Table 1, the majority of oxylipins were present in cortex, and many that were not detected in MW-fixed control brains, were present upon ischemia.

**Table 1:** Number of oxylipins detected in cortex, hippocampus, cerebellum and brainstem in the control (n = 7–9) and ischemic groups (n = 7–9 per group) relative to the total number of metabolites analyzed (in brackets).

	Cortex		Hippocampus		Cerebellum		Brainstem	
	Control	Ischemic	Control	Ischemic	Control	Ischemic	Control	Ischemic
LA-derived metabolites	11 (/12)	11 (/12)	6 (/12)	8 (/12)	9 (/12)	9 (/12)	7 (/12)	9 (/12)
DGLA-derived metabolites	2 (/3)	2 (/3)	0 (/3)	2 (/3)	0 (/3)	2 (/3)	0 (/3)	2 (/3)
AA-derived metabolites	8 (/34)	27 (/34)	4 (/34)	17 (/34)	5 (/34)	21 (/34)	6 (/34)	17 (/34)
ALA-derived metabolites	2 (/8)	2 (/8)	0 (/8)	0 (/8)	1 (/8)	4 (/8)	2 (/8)	2 (/8)
EPA-derived metabolites	2 (/16)	2 (/16)	0 (/16)	1 (/16)	1 (/16)	1 (/16)	0 (/16)	1 (/16)
DHA-derived metabolites	5 (/12)	9 (/12)	6 (/12)	6 (/12)	5 (/12)	6 (/12)	6 (/12)	6 (/12)
Total metabolites	30 (/85)	53 (/85)	16 (/85)	34 (/85)	21 (/85)	43 (/85)	21 (/85)	37 (/85)

**Figure 1:**
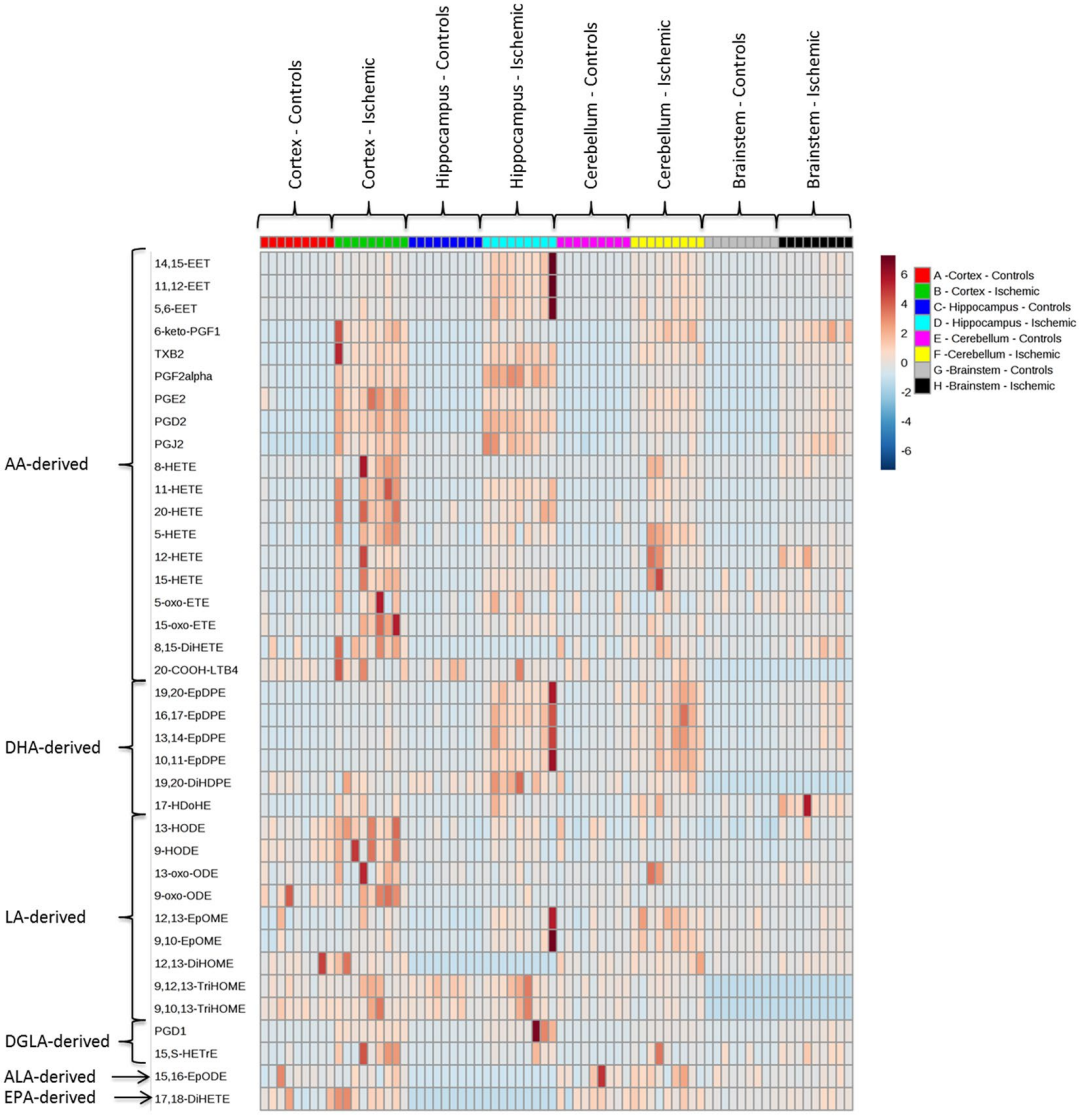
Heat map of oxylipin concentrations in cortex, hippocampus, cerebellum and brainstem in control and ischemic rats. EET, epoxyeicosatrienoic acid; PG, prostaglandin; TXB2, Tromboxane B2; HETE, hydroxyeicosatetraenoic acid; oxo-ETE, oxo-eicosatetraenoic acid; DiHETE, dihydroxyeicosatetraenoic acid; LTB4, leukotriene B4; EpDPE, epoxydocosapentaenoic acid; DiHDPE, dihydroxydocosapentaenoic acid; HDoHE, hydroxydocosahexaenoic acid; HODE, hydroxyoctadecadienoic acid; oxo-ODE, oxo-octadecadienoic acid; EpOME, epoxyoctadecamonoenoic acid; diHOME, dihydroxyoctadecamonoenoic acid; TriHOME, trihydroxyoctadecamonoenoic acid; EpODE, epoxyoctadecadienoic acid; HETrE, hydroxyeicosatrienoic acid.

A heat map depicting the oxylipins common to all 4 brain regions in control and ischemic brains is shown in Fig. 1. As indicated, many LA, AA and DHA metabolites were abundant in all brain regions at baseline, and increased markedly following ischemia. Interestingly, although LA itself is low in brain compared to AA and DHA^28^, its metabolites were abundant.

### Ischemia increased LA-derived metabolites in various brain regions

**Figure 2:**
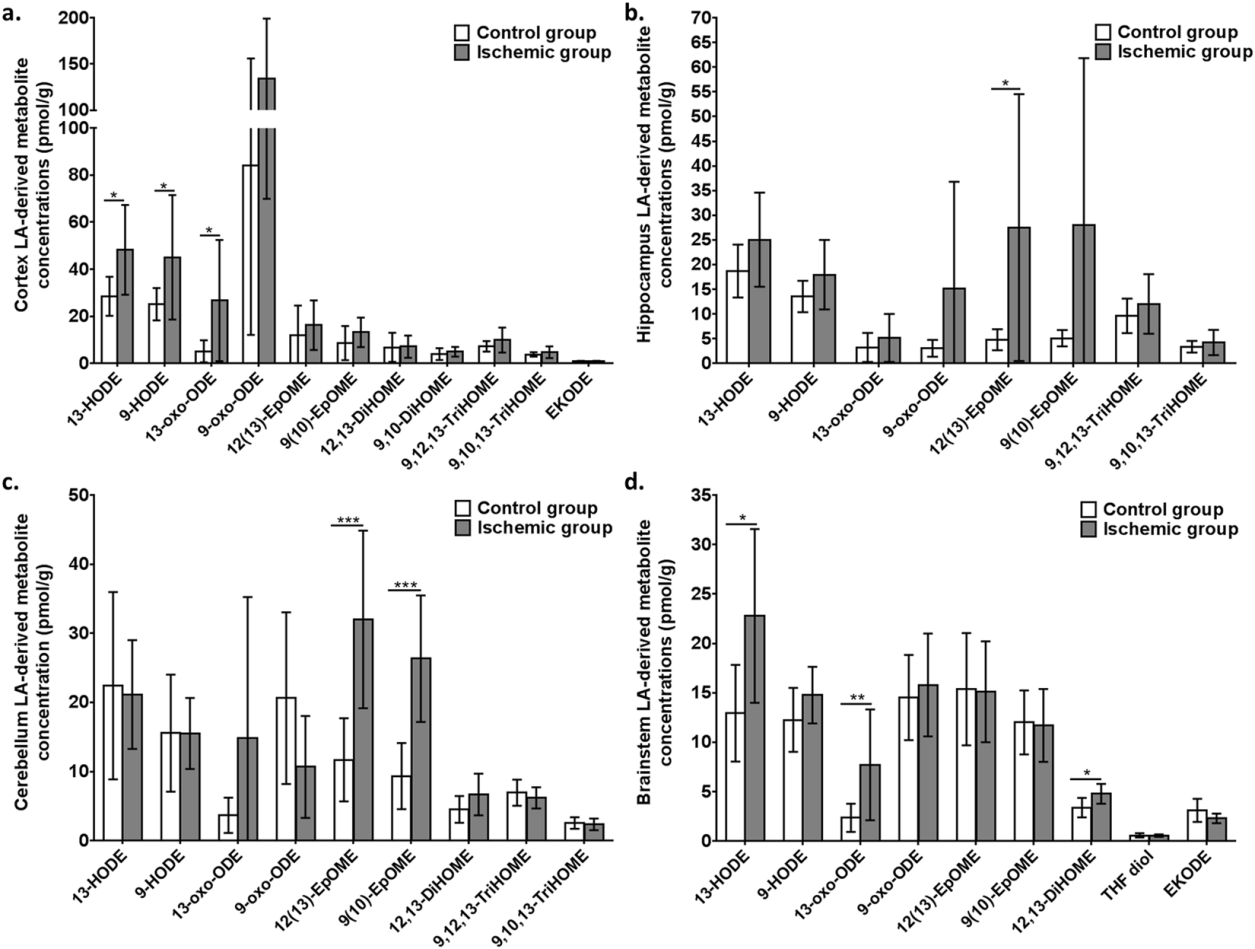
Cortex (**a**), hippocampus (**b**), cerebellum (**c**) and brainstem (**d**) linoleic acid (LA)-derived metabolite concentrations (in pmol/g) in microwave (MW) control and ischemic rats (CO_2_; n = 7–9 per group). Values are mean ± standard deviation (SD). Significant differences were assessed using an unpaired t-test (*p < 0.05; **p < 0.01; ***p < 0.001). HODE, hydroxyoctadecadienoic acid; oxo-ODE, oxo-octadecadienoic acid; EpOME, epoxyoctadecamonoenoic acid; DiHOME, dihydroxyoctadecamonoenoic acid; TriHOME, trihydroxyoctadecamonoenoic acid; THF, tetrahydrofuran; EKODE, epoxyketooctadecadienoic acid.

LA-derived oxylipins were significantly increased in various brain regions following ischemia compared to MW-fixed controls (Fig. 2). 13-HODE was 1.7-fold higher in the ischemic CO_2_-group compared to MW-fixed controls in cortex (*p* = 0.0115) and brainstem (*p* = 0.0098). 9-HODE was also increased in cortex by 1.8-fold in the ischemic CO_2_-group compared to MW-fixed controls (*p* = 0.0439). 13-oxo-ODE was increased by 5.6-fold in cortex (*p* = 0.0499) and by 3.2-fold in brainstem (*p* = 0.0134). 12(13)-EpOME was increased in both hippocampus (5.7-fold; *p* = 0.023) and cerebellum (2.7; *p* < 0.001), whereas 9(10)-EpOME was increased 2.8-fold in cerebellum (*p* < 0.001) of ischemic rats compared to MW-fixed controls. Brainstem concentrations of 12,13-DiHOME increased by 1.4-fold (*p* = 0.0088), following CO_2_-induced ischemia.

### Ischemia increased AA-derived metabolites in various brain regions

**Figure 3:**
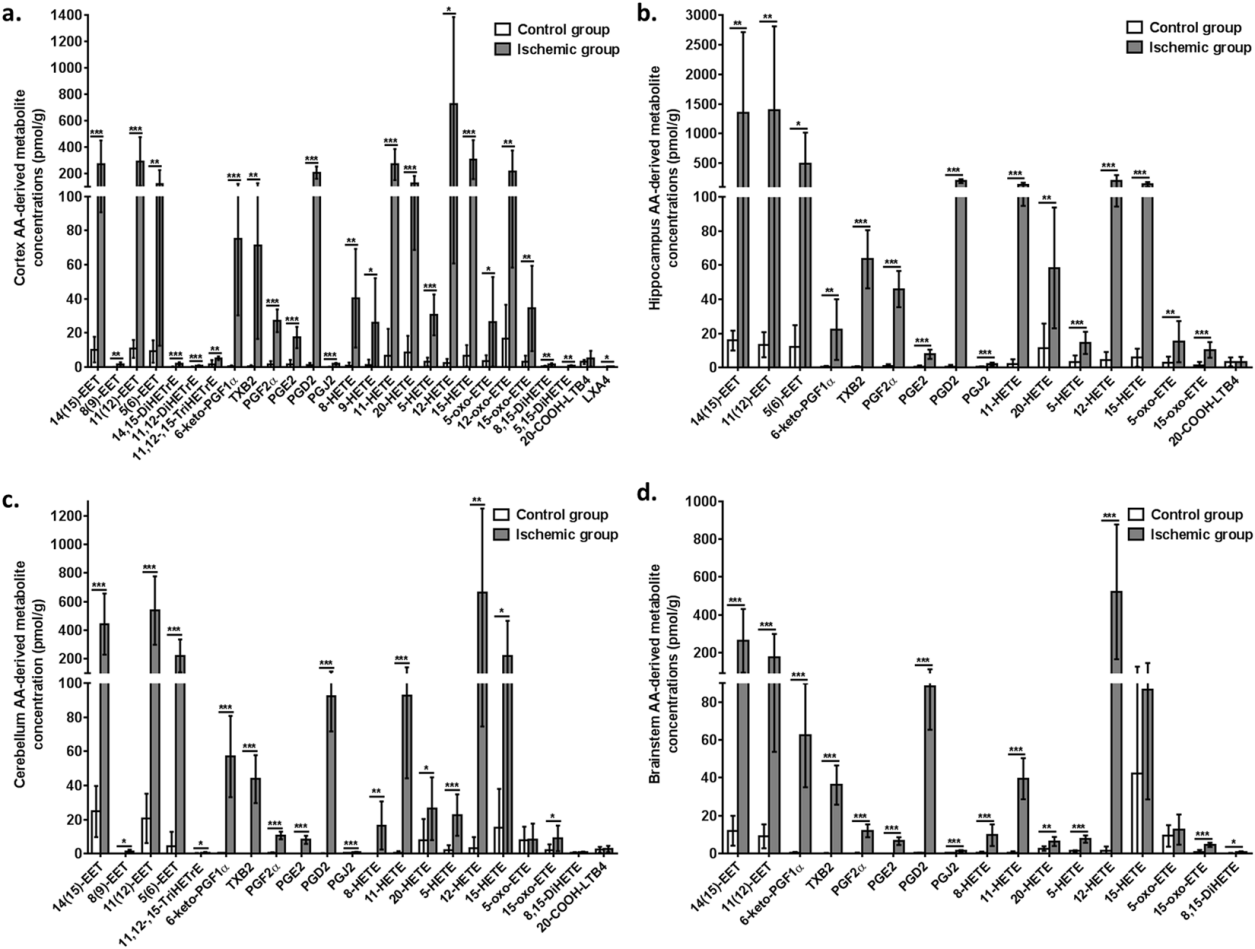
Cortex (**a**), hippocampus (**b**), cerebellum (**c**) and brainstem (**d**) arachidonic acid (AA)-derived metabolite concentrations (in pmol/g) in microwave (MW) control and ischemic rats (CO_2_; n = 7–9 per group). Values are mean ± standard deviation (SD). Significant differences were assessed using an unpaired t-test (*p < 0.05; **p < 0.01; ***p < 0.001). EET, epoxyeicosatrienoic acid; DiHETrE, dihydroxyeicosatrienoic acid; TriHETrE, trihydroxyeicosatrienoic acid; PG, prostaglandin; TXB2, thromboxane B2; HETE, hydroxyeicosatetraenoic acid; oxo-ETE, oxo-eicosatetraenoic acid; DiHETE, dihydroxyeicosatetraenoic acid; LTB4, leukotriene B4; LXA4, lipoxins A4.

**Figure 4:**
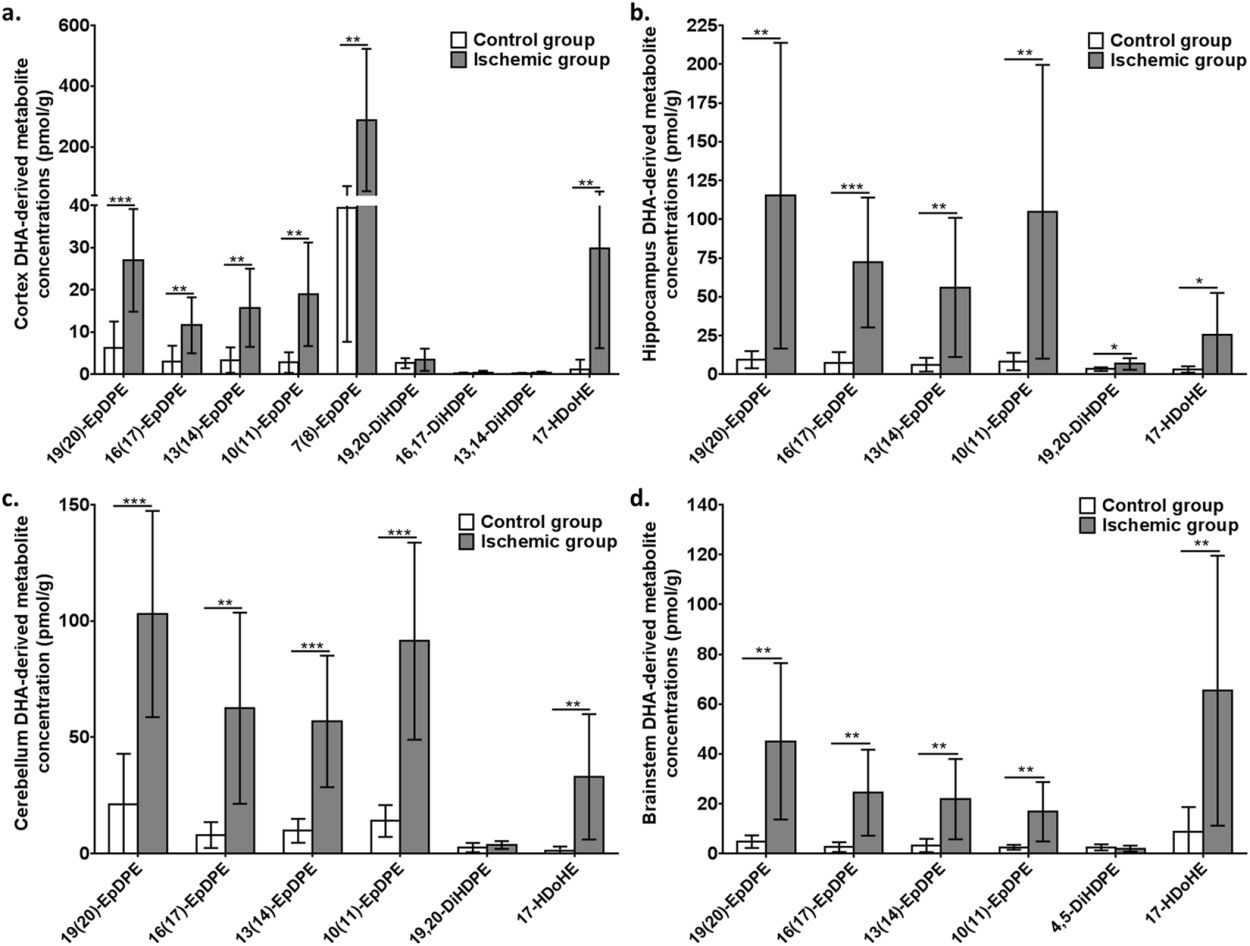
Cortex (**a**), hippocampus (**b**), cerebellum (**c**) and brainstem (**d**) docosahexaenoic acid (DHA)-derived metabolite concentrations (in pmol/g) in microwave (MW) control and ischemic rats (CO_2_; n = 7–9 per group). Values are mean ± standard deviation (SD). Significant differences were assessed using an unpaired t-test (*p < 0.05; **p < 0.01; ***p < 0.001). EpDPE, epoxydocosapentaenoic acid; DiHDPE, dihydroxydocosapentaenoic acid; HDoHE, hydroxydocosahexaenoic acid.

Previous studies reported an increase in the formation of AA- and DHA- derived oxylipins following hypoxia or ischemic brain injury^41, 49^. To confirm that these changes occurred in the present study, regional changes in AA- and DHA- derived metabolites were measured by LC-MS/MS as shown in Figs 3 and 4, respectively.

Compared to the MW-fixed controls, AA-derived epoxy-metabolites, 14(15)-EET, 11(12)-EET and 5(6)-EET, were increased in cortex (13 to 55-fold; *p* < 0.01), hippocampus (41 to 104-fold; *p* < 0.05), cerebellum (18 to 52-fold; *p* < 0.05) and brainstem (19 to 22-fold; *p* < 0.001) of the ischemic CO_2_-group. Mono-hydroxylated AA-derived metabolites, 5-, 11-, 12-, 15- and 20-HETE, were increased in cortex (10 to 345-fold; *p* < 0.05), hippocampus (4 to 67-fold; *p* < 0.01), cerebellum (3 to 204-fold; *p* < 0.05) and brainstem (2.5 to 372-fold; *p* < 0.001), following ischemia. 15-oxo-ETE, an AA-derived ketone, was increased by 11-fold in cortex (*p* = 0.0067), 7.4-fold in hippocampus (*p* < 0.0001), 4.7-fold in cerebellum (*p* = 0.03) and 5.2-fold in brainstem (*p* < 0.0001), while 5-oxo-ETE was increased by 7.9-fold in cortex (*p* = 0.039) and 5.3-fold in hippocampus (*p* = 0.0099), and 12-oxo-ETE by 12.9-fold in cortex (*p* = 0.0061) (Fig. 3).

Prostanoids (6-keto-PGF_1α_, PGF_2α_, PGE_2_, PGD_2_, PGJ_2_) were negligible or not detected in MW-fixed controls, but were present in the four brain regions of CO_2_-treated rats (*p* < 0.001). Ischemia also increased the cortical concentrations of epoxy- (8(9)-EpEtrE; *p* = 0.0048), monohydroxy- (8- and 9- HETE; *p* < 0.05), dihydroxy- (14,15- and 11,12-DiHETrE, *p* < 0.001; 8,15- and 5,15-DiHETE, *p* < 0.01) and trihydroxy- (11,12,15-TriHETrE; *p* = 0.0012) AA metabolites, which were not detected in MW-fixed controls (Fig. 3).

### Ischemia increased DHA-derived metabolites in various brain regions

DHA-derived epoxy-metabolites (19(20)-, 16(17)-, 13(14)-, 10(11)- and 7(8)-EpDPE) were higher in cortex (3.9 to 7.3-fold; *p* < 0.01), hippocampus (9 to 12.7-fold; *p* < 0.01), cerebellum (4.8 to 7.9-fold; *p* < 0.01) and brainstem (6.6 to 9.4-fold; *p* < 0.01) of ischemic rats compared to MW-fixed controls (Fig. 4). 17-HDoHE was detected in cortex and cerebellum (*p* < 0.01) of ischemic CO_2_-treated rats but not MW-fixed controls, and was 8.1- and 7.4-fold higher in hippocampus (*p* = 0.025) and brainstem (*p* = 0.0072) of ischemic rats, respectively, relative to controls. Amongst the dihydroxy DHA metabolites analyzed, only 19,20-DiHDPE increased by 2-fold in hippocampus of the ischemic CO_2_-group compared to MW-fixed controls (*p* = 0.021).

### Other fatty acid metabolites found in relatively low concentrations in control brains were increased following ischemia

**Figure 5:**
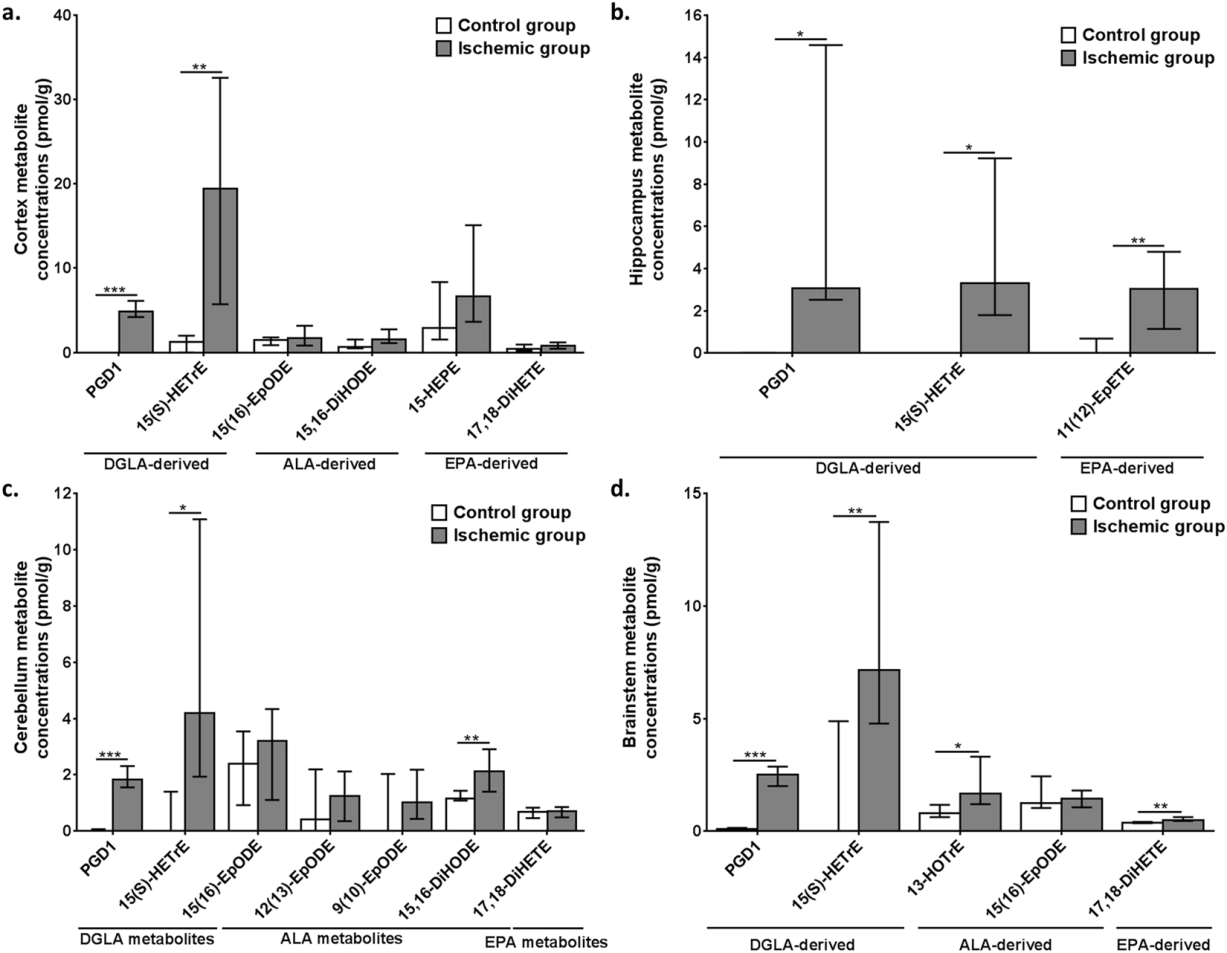
Cortex (**a**), hippocampus (**b**), cerebellum (**c**) and brainstem (**d**) di-homo-gamma-linolenic acid (DGLA)-, α-linolenic acid (ALA)- and eicosapentaenoic acid (EPA)-derived metabolite concentrations (in pmol/g) in microwave (MW) control and ischemic rats (CO_2_; n = 7–9 per group). Values are mean ± standard deviation (SD). Significant differences were assessed using an unpaired t-test (*p < 0.05; **p < 0.01; ***p < 0.001). PG, prostaglandin; HETrE, hydroxyeicosatrienoic acid; EpODE, epoxyoctadecadienoic acid; DiHODE, dihydroxyoctadecadienoic acid; HEPE, hydroxyeicosapentaenoic acid; DiHETE, dihydroxyeicosatetraenoic acid; EpETE, epoxyeicosatetraenoic acid; HOTrE, hydroxyoctadecatrienoic acid.

The concentrations of DGLA, ALA and EPA-derived metabolites within the different brain regions were low and only few of them were detected (Fig. 5). DGLA-derived PGD_1_ and 15(S)-HETrE, were present in all brain regions of the ischemic CO_2_-group but were absent or negligible in the MW-fixed group (*p* < 0.01). ALA-derived 15,16-DiHODE was 2.3 and 1.9 times higher in cortex (*p* = 0.0314) and cerebellum (*p* = 0.0028) of ischemic CO_2_-rats relative to MW-fixed controls, respectively. ALA-derived 13-HOTrE increased by 1.9 fold in brainstem (*p* = 0.0078). EPA-derived 11(12)-EpETE was detected in hippocampus following ischemia, but not in controls. Other detected EPA-derived metabolites did not significantly differ between the groups.

### 13-HODE and PGE_2_, but not their fatty acid precursors, increased somatic PPF

Hippocampal extracellular recordings were performed to test whether 13-HODE, the main LA metabolite detected in hippocampus (Fig. 2), altered neurotransmission in a manner comparable to its precursor, LA, and to AA and AA-derived PGE_2_.

**Figure 6:**
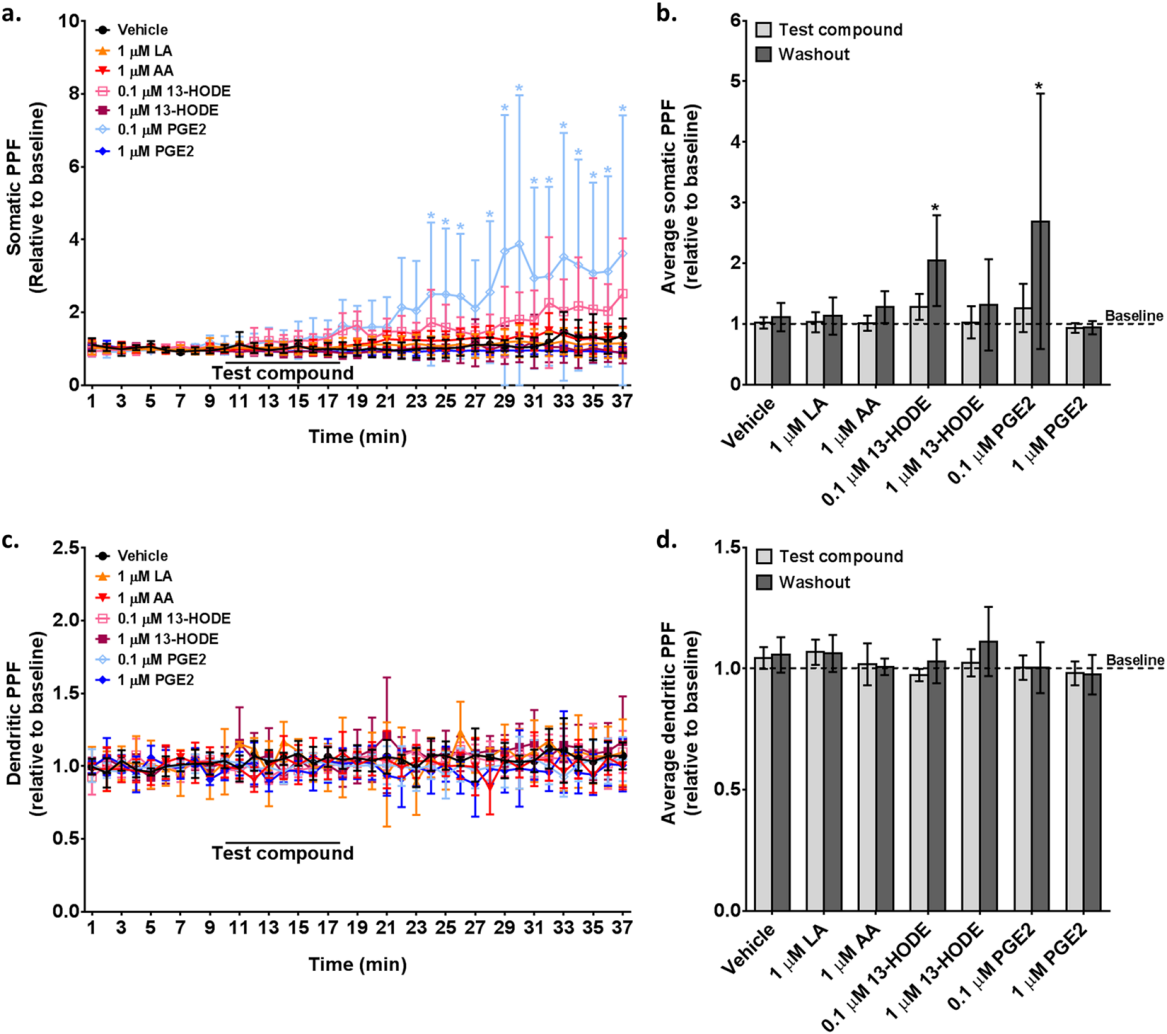
Somatic (**a** and **b**) and dendritic (**c** and **d**) Paired-Pulse Facilitation (PPF) measured on hippocampal slices perfused with vehicle (artificial cerebrospinal fluid containing 0.1% ethanol), 1 µM linoleic acid (LA), 1 µM arachidonic acid (AA), 0.1 µM or 1 µM 13-hydroxyoctadecadienoic acid (13-HODE), or 0.1 µM or 1 µM prostaglandin E_2_ (PGE_2_). Data (mean ± SD) are expressed relative to baseline (n = 4–6 per condition). Graphs (**a** and **c**) showe the minute-by-minute data; (**b** and **d**) represent average PPF during compound incubation and washout relative to baseline (dotted line). Data were analyzed by a two-way repeated measures ANOVA followed by Dunnett’s multiple comparison test. *Significantly different compared to vehicle at a specific time point.

Two-way repeated measures ANOVA revealed a significant effect of time (*p* < 0.0001), and time-compound interaction (*p* < 0.0001) on the minute-by-minute PPF in soma. As indicated in Fig. 6a, compared to vehicle, 0.1 µM PGE_2_ significantly increased somatic PPF during the washout period (at 24, 25, 26 and after 28 minutes of recording; *p* < 0.05 by Dunnett’s post-hoc test). Mean somatic PPF was also significantly altered by time (*p* = 0.0003), compound (*p* = 0.0287) and time-compound interaction (*p* = 0.0121), as evidenced by the significant 1.8-fold and 2.4-fold increase in somatic PPF by 0.1 µM 13-HODE (*p* = 0.0296) and 0.1 µM PGE_2_ (*p* < 0.0001) during the washout period (Fig. 6b). No significant effects of time or treatment were observed in dendritic PPF (Fig. 6c and 6d).

**Figure 7:**
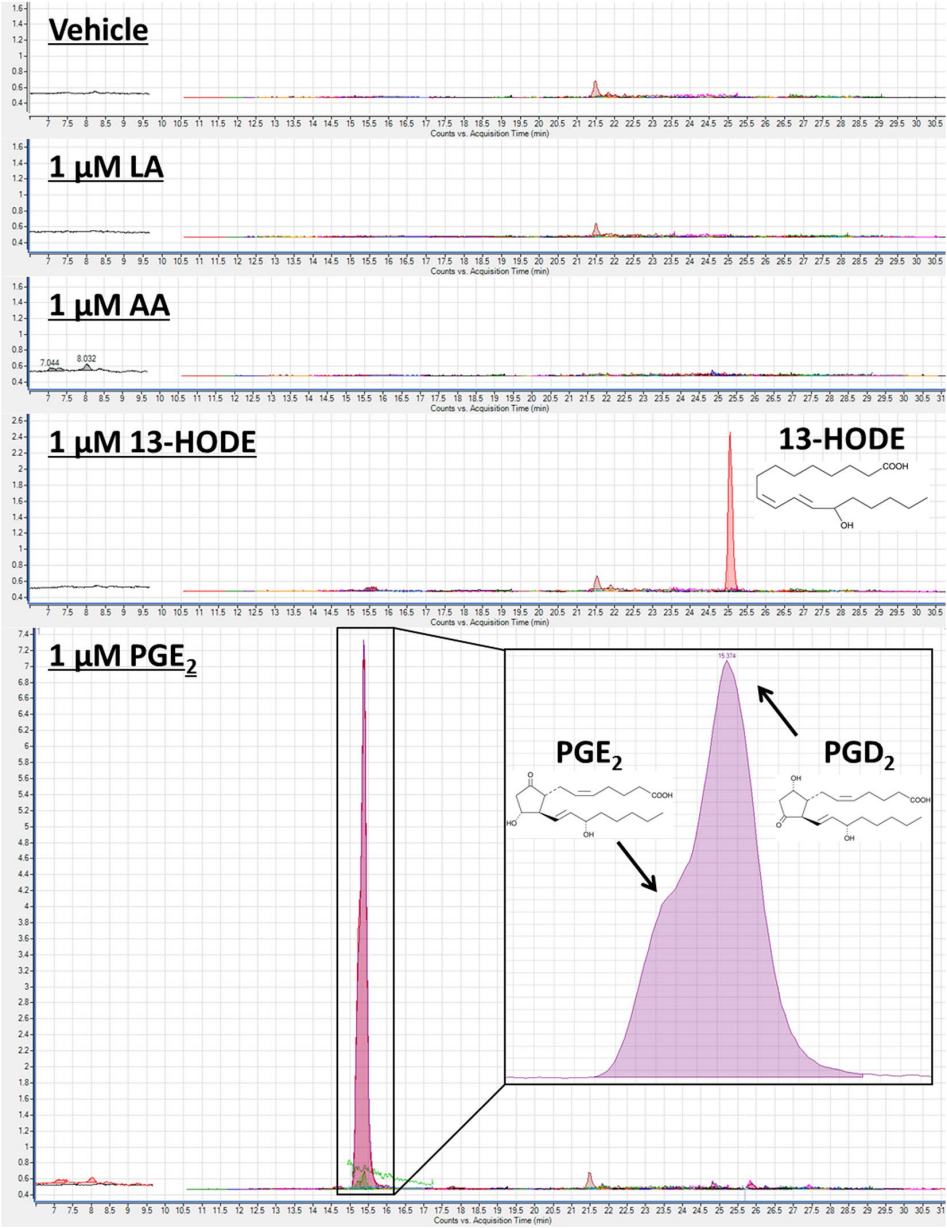
Dynamic multiple reaction monitoring scan of oxidized fatty acids in artificial cerebrospinal fluid (ACSF) containing vehicle, 1 µM linoleic acid (LA), 1 µM arachidonic acid (AA), 1 µM 13-hydroxyoctadecadienoic acid (13-HODE), or 1 µM prostaglandin E_2_ (PGE_2_). The vehicle or compounds were incubated in ACSF at 37 °C for 10 min under constant bubbling of 95% O_2_. No contamination was observed in vehicle and ACSF containing LA and AA. ACSF containing 13-HODE was pure at >98%. ACSF containing PGE_2_ had 20% PGE_2_, 78% PGD_2_ and 2% unidentified impurities. As shown in the figure, PGE_2_ and PGD_2_ peaks eluted at the same time.

Gas-chromatography analysis confirmed the purity of LA and AA stock solutions to be 96.6% and 91.7%, respectively. AA contained a small amount of palmitic acid (C16:0; 1.3%), oleic acid (C18:1 n-9; 1.0%) and LA (0.3%). LC-MS/MS analysis showed that 13-HODE and PGE_2_ were 98–99% pure in the stock solution. LC-MS/MS analysis of artificial cerebrospinal fluid (ACSF) aliquots obtained at the end of the 10-minute perfusion was also performed to test whether the fatty acids or oxylipins were degraded during their incubation in ACSF at 37 °C under constant bubbling of 95% oxygen. As shown in Fig. 7, AA and LA did not degrade into any of the measured oxylipins, although this does not preclude the possibility of degradation into other compounds not covered by our lipidomic assay such as AA-derived F2-isoprostanes. ACSF aliquots of 13-HODE were >99% pure. PGE_2_, however, contained 20% PGE_2_ and 78% PGD_2_, suggesting degradation of the PGE_2_ into PGD_2_ during the 10-minute perfusion period.

## Discussion

Here, we provide new evidence that LA is involved in the response to ischemia-induced brain injury and the regulation of neurotransmission through its oxidized metabolites. Ischemia increased cortex, cerebellum, hippocampus and brainstem LA-derived oxylipin concentrations, of which 13-HODE was tested and found to increase somatic PPF in hippocampus similar to AA-derived PGE_2_. The results suggest that during ischemic brain injury, the brain actively produces LA-metabolites that regulate neuronal signaling.

The present study confirmed previous reports of increased AA-derived prostaglandins (in particular PGE_2_ and PGD_2_), thromboxane B2 and lipoxygenase products (HETE, oxo-ETE and leukotrienes)^41, 46, 47, 56, 70^, and increased DHA-derived 17-HDoHE^41, 49^ in hippocampus or whole brain of rodents subjected to hypoxic or ischemic brain injury. By incorporating an expanded AA and DHA oxylipin panel in our LC-MS/MS platform, we also found an increase in epoxidized metabolites of AA and DHA following ischemia in cortex, hippocampus, cerebellum and brainstem. Because AA- and DHA-derived epoxides are anti-inflammatory and neuroprotective^71–74^, their increase following ischemia likely represents an adaptive response to prevent ischemia-related brain injury.

The parallel increase in LA metabolites in cortex, hippocampus, cerebellum and brainstem suggests that LA oxidized products are also involved in the response to ischemic brain injury, consistent with one study that reported increased 9- and 13-HODE in cortex of dogs subjected to 10-minutes of ischemic cardiac arrest^75^. CYP-derived LA epoxides (9(10)- and 12(13)-EpOME) were increased in hippocampus and cerebellum, two brain regions sensitive to hypoxic and ischemic insults^58–61^, whereas LOX-derived 9- and 13-HODE and oxo-ODE were increased in cortex and the brainstem. This reflects the selective synthesis of LA-derived species that likely play diverse roles during ischemia.

Many oxylipins produced following brain injury (prostanoids or epoxides) are known to regulate neurotransmission coupled to physiological processes that promote vasodilation or reduce excitotoxicity^48, 49, 76^. In the present study, we tested whether LA-derived 13-HODE also regulated neurotransmission. 13-HODE at 0.1 µM increased somatic but not dendritic paired-pulse facilitation in hippocampus, suggesting its involvement in regulating post-synaptic transmission and consistent with the somato-dendritic localization of COX and LOX enzymes that rapidly synthesize it^77^. 13-HODE was found to block phospholipase C-induced activation of protein kinase C^78^, a key regulator of short-term plasticity^79^. The mechanism of action of 13-HODE may involve a G-protein coupled receptor, such as the G2A receptor which binds oxidized fatty acid metabolites^80^. Identifying the specific G-protein receptor(s) that selectively binds 13-HODE in future studies might elucidate the mechanisms by which 13-HODE regulates neurotransmission in response to ischemic brain injury. Confirming that 13-HODE also regulates neurotransmission in cortex and brainstem, two brain regions were 13-HODE increased during ischemia, will inform on whether 13-HODE acts globally or on specific brain regions.

13-HODE and PGE_2_ increased somatic PPF at 0.1 µM but not 1 µM. The 0.1 µM dose of 13-HODE and PGE_2_ is consistent with the amount found in brain (based on measured concentrations in Figs 2 and 3 corrected for brain density), thus being physiologically relevant. Hippocampal dendritic PPF was reported to decrease by 5 µM^67, 68^ or remain unchanged by 0.5 µM^81^ or 10 µM^69^ PGE_2_, respectively. We are not aware of studies that specifically explored the effects of PGE_2_ on somatic transmission. However, by quantifying PGE_2_ (and 13-HODE) in hippocampus, this study demonstrated the signaling effects of both compounds at physiologically relevant concentrations and showed that higher doses were ineffective. Unesterified LA and AA did not alter PPF when applied at a physiologically relevant concentration of 1 µM^82^, suggesting that their signaling effects in brain are likely mediated by their metabolites.

Approximately 78% of the PGE_2_ was converted to PGD_2_ in the ACSF chamber, before reaching the slice. This means that the observed changes in hippocampal PPF in this study and possibly others^67–69^ could be mediated by PGD_2_. Little is known about the role of PGD_2_ on hippocampal neurotransmission. Chen *et al*. reported that 0.33 µM PGD_2_ did not alter postsynaptic excitability and induction of long-term potentiation in the presence of a COX-2 inhibitor, suggesting it likely has limited effects on neuronal excitability^83^.

Regional increases in brain EPA-derived 11,12-EpETE and 17,18-diHETE, ALA-derived 13-HoTrE and DGLA-derived PGD_1_ and 15(S)-HETrE were also seen following ischemia. DGLA-, ALA- and EPA- derived metabolites have been reported to reduce inflammation *in vitro* and *in vivo* ^84–87^, although their role in regulating neurotransmission or the response to brain injury is not known. The observed increase in their concentrations following ischemia highlights the need to explore their neurophysiological role and bioactivity in future studies.

In summary, this study showed that LA participates in the response to ischemic brain injury through metabolites that also regulate neurotransmission. Targeting this pathway using low LA diets^8, 31^ or novel drugs may be therapeutically useful for ischemia-related conditions such as stroke or hypoxic-ischemic encephalopathy of newborn infants.

## Methods

### Animals

All procedures were performed in agreement with the policies of the Canadian Council on Animal Care and were approved by the Animal Ethics Committee of the University of Toronto and University Health Network. Thirty to thirty-four day old male rats were purchased from Charles River (Saint-Constant, QC, Canada). Upon their arrival, rats were housed in pairs and fed for 30 days with a Harlan Teklad 2018 diet containing 18.6% protein, 6.2% fat, 58.9% carbohydrate, 3.5% crude fiber and 5.3% ash and 7.5% moisture. The diet contained (% of total fatty acids), 18.5% palmitic acid (16:0), 2.8% stearic acid (18:0), 18.5% oleic acid (18:1 n-9), 54.8% LA and 5.6% ALA^88^.

### Rat tissue collection

Rats were subjected to head-focused microwave (13.5 kW for 1.6 s; MW-fixed control group; n = 9) or CO_2_ asphyxiation for 2 min (CO_2_-group; n = 9)^89^. Brains were rapidly removed following head decapitation and separated into cortex, cerebellum, hippocampus and brainstem on ice. The use of CO_2_ causes hypercapnia, which is followed by decapitation-induced ischemia. The effects of hypercapnia on measured oxylipins are minimal compared to that of ischemia, which is why the effects reported in this study will be linked to ischemia rather than the combined effect of hypercapnia and ischemia^89^. Samples were stored at −80 °C for approximately one month until they were shipped on dry ice from Toronto, ON, Canada to Davis, CA, USA, where they were stored in a −80 °C freezer until use.

### Oxylipin extraction by solid phase extraction (SPE)

Oxylipins were extracted from the different cerebral regions by solid phase extraction (SPE), as previously described^31, 90, 91^. Two to four hundred µL of ice-cold extraction solvent (0.1% acetic acid and 0.1% butylated hydroxytoluene (BHT) in methanol) was added to frozen cortex (average weight >200 mg; 400 µL of extraction solvent), hippocampus, cerebellum and brainstem (weight <200 mg; 200 µL of extraction solvent), followed by the addition of 10 µL of antioxidant mix and 10–20 µL surrogate standard solution. The antioxidant solution containing 0.2 mg/mL of BHT, triphenylphosphine (TPP) and ethylenediaminetetraacetic acid (EDTA) in methanol/water (50/50, v/v) was filtered through a Millipore filter (Millipore, Bedford, MA, USA) prior to use. The surrogate standard solution contained 500 nM of d11–11(12)-EpETrE, d11-14,15-DiHETrE, d4-6-keto-PGF_1α_, d4-9-HODE, d4-LTB_4_, d4-PGE_2_, d4-TXB_2_, d6-20-HETE and d8-5-HETE in methanol, and was added at an amount of 5–10 pmol per sample.

Frozen pre-weighed samples were homogenized for 5 to 10 min at 30 vibrations per second with a bead homogenizer. After storage overnight in a −80 °C freezer, samples were centrifuged at 13,200 rpm for 10 min at 4 °C. Two hundred µL of supernatant were added to a 60 mg Waters Oasis HLB 3cc cartridges (Waters, Milford, MA, USA), pre-rinsed with one volume of ethyl acetate and two volumes of methanol, and pre-conditioned with two volumes of SPE buffer containing 5% methanol and 0.1% acetic acid in ultrapure water. The columns were rinsed twice with SPE buffer and dried under vacuum (≈20 psi) for 20 min. Oxylipins were eluted with 0.5 mL methanol and 1.5 mL ethyl acetate into a 2 mL centrifuge tube containing 6 µL of glycerol in methanol (30%).

Samples were dried in a Speed-Vac®, reconstituted in 50 µL methanol containing 200 nM 1-cyclohexyl ureido, 3-dodecanoic acid (CUDA) as a recovery standard and filtered by centrifugation using Ultrafree-MC-VV polyvinylidene fluoride filters (0.1 µm; Millipore, Bedford, MA, USA).

### Oxylipin analysis by liquid chromatography tandem mass spectrometry (LC-MS/MS)

The PUFA-derived oxylipin analytical platform included 85 oxylipins (Supplementary Table 1) derived from omega-6 LA, LA’s elongation-desaturation products DGLA and AA, and omega-3 ALA, EPA and DHA. Oxylipins were analyzed by ultra-high pressure liquid chromatography tandem mass spectrometry UPLC-MS/MS as previously described^90, 92^ on an Agilent 1200SL (Agilent Corporation, Palo Alto, CA, USA) UPLC system connected to a 4000 QTRAP tandem mass spectrometer (Applied Biosystems Instrument Corporation, Foster, CA, USA) equipped with an electrospray ion source (Turbo V). Oxylipins were separated on an Agilent 2.1 × 150 mm Eclipse Plus C18 column with a 1.8 µm particle size. Standards obtained from Larodan (Solna, Sweden), Cayman Chemicals (Ann Arbor, MI, USA) or synthesized by Dr. Hammock’s laboratory were used for calibration curves for each oxylipin.

The autosampler temperature was kept at 4 °C and the column at 50 °C. The mobile phase A contained 0.1% acetic acid in ultrapure water and the mobile phase B contained acetonitrile/methanol /acetic acid (84/16/0.1). Gradient elution was performed at a flow rate of 0.25 mL/min for a total run time of 21 min as follows: solvent B was held at 35% for 0.25 min, increased to 45% from 0.25 to 1 min, to 55% B from 1 to 3 min, to 65% B from 3 to 8.5 min, to 72% from 8.5 to 12.5 min, to 82% B from 12.5 to 15 min, to 95% B from 15 to 16.5 min, held at 95% for 1.5 min, decreased to 35% from 18 to 18.1 min and held at 35% for 2.9 min. The instrument was operated in negative electrospray ionization mode and used optimized multiple reaction monitoring (MRM) conditions of the parent and fragmentation product ion to target each oxylipin^90^. Peaks were quantified according to the standard curves and corrected for the surrogate standard recovery using Analyst software 1.4.2.

The limit of quantification (LOQ) was set to three times the lowest standard concentration used in the standard curve. Oxylipins with >30% of values below the LOQ were excluded from the statistical analysis.

### Preparation of the artificial cerebrospinal fluid (ACSF) solutions

A low Na^+^/Ca_2_^+^ ACSF containing 50 mM NaCl, 160 mM sucrose, 3.5 mM KCl, 2 mM NaH_2_PO_4_, 0.5 mM CaCl_2_, 2 mM MgCl_2_, 7 mM glucose and 5 mM HEPES (pH adjusted to 7.4) was prepared and used during intracardial perfusion and the slice preparation to limit excitotoxicity. A standard ACSF containing 3.5 mM KCl, 1.25 mM NaH_2_PO_4_, 125 mM NaCl, 25 mM NaHCO_3_, 10 mM glucose, 2 mM CaCl_2_ and 1.3 mM MgSO_4_ (pH 7.4 when aerated with 95% O_2_−5% CO_2_) was used during the hippocampal slice perfusion and electrophysiology recordings.

### Slice preparation

Experiments were performed on 400-µm-thick hippocampal slices from 62- to 74-d-old male Long Evans rats (Charles River Laboratory, Quebec, Canada). Rats were euthanized with a lethal dose of sodium pentobarbital (70 mg/kg) and intracardially perfused with cold low Na^+^/Ca_2_^+^ ASCF. After decapitation, the brain was rapidly removed and maintained in ice-cold oxygenated (95% O_2_−5% CO_2_) low Na^+^/Ca_2_^+^ ACSF for a few minutes. The brain was hemi-sectioned and the hippocampus isolated and glued onto an aluminum block. Four hundred-µm-thick transverse hippocampal slices were obtained using a vibratome and then placed in the standard ACSF at room temperature for at least 1 hour before recordings.

### Extracellular recordings

Extracellular recordings were used to test the isolated effect of each compound on paired-pulse facilitation. Extracellular recordings were obtained from 4 to 6 slices per fatty acid or oxylipin treatment. Slices were transferred to a submerged recording chamber and continuously perfused with warm (37 °C) oxygenated (95% O_2_−5% CO_2_) standard ACSF at a flow rate of 10 mL/min. All recordings were done at a perfusate temperature of 37 °C. The Schaeffer collateral pathway was stimulated electrically with a bipolar stimulating electrode (polyamide-insulated stainless steel wires; outer diameter 100 μm; Plastics One, Ranoake, VA) placed in the stratum radiatum at the CA1-CA2 border. Recording electrodes were made from thin wall glass tubes (OD 1.5 mm; ID 1.12 mm; World precision Instruments, Sarasota, FL) filled with ACSF and placed in the stratum pyramidale (soma) and stratum radiatum (dendrite) of the CA1 region. Constant-current pulses (duration of 0.1 ms each, intensities of 10–150 μA) were generated by a Grass stimulator (model S88, Grass Medical Instruments, Warwick, RI, USA) and delivered through an isolation unit. Extracellular signals were recorded using a dual channel amplifier (700B) and digitized using an analog-digital converter (Digidata 1400, Molecular Devices, Sunnyvale, CA, USA). Data acquisition, storage and analysis were done using the pCLAMP software (version 10.5, Molecular Devices, Sunnyvale, CA, USA).

To examine paired-pulse facilitation (PPF), twin stimuli (intensity range: 10–100 μA) were delivered with an interpulse interval of 35 ms. Representative recordings are shown in Supplementary Figure 1. Paired-stimuli were delivered every 10 s. Baseline recordings were measured for at least 10 min, followed by compound delivery for 8–15 min and finally, a washout period of at least 19 min (n = 4–6 per condition). The somatic amplitudes and dendritic field postsynaptic potential slopes were measured. PPF was calculated every minute by taking the ratio of the second response to the first response.

### Compounds

13-HODE and PGE_2_ were purchased from Cayman Chemicals (Ann Arbor, Michigan, USA). LA and AA were purchase from Nuchek Prep, Inc. (Elysian, MN, USA). The different drugs were dissolved in ethanol (stock concentration at 1 mM or 0.1 mM) and diluted 1000 times in ACSF to 1 µM LA, 1 µM AA, 0.1 µM or 1 µM 13-HODE, and 0.1 µM or 1 µM PGE_2_. Vehicle was made by diluting 100 µL of pure ethanol per 100 mL ACSF. The final ethanol concentration for vehicle or compounds was kept at or below 0.1%. The fatty acid precursors, LA and AA, were tested at 1 µM to mimic physiological conditions, because brain unesterified LA and AA concentrations in rodents range between 1–3 nmol/g^28^, which corresponds to 0.96–2.88 µM, based on a rat brain density of 1.04–1.05 g/mL^93^.

In an exploratory manner, we also tested the effects of AA-derived 14(15)-EET synthetized to 99% purity, at 1 µM and of LA-derived 9-oxo-ODE at 0.1 µM (Cayman Chemicals). We had intended to test lower concentrations of 14(15)-EET, but by testing the dose of 1 µM, we ran out of the compound and were not able to perform tests at 0.1 µM. Pilot data related to the effects of 14(15)-EET on PPF are provided in Supplementary Figure 2. As shown, 14,15-EET significantly reduced dendritic PPF during the washout period. The 9-oxo-ODE data were not included because LC-MS/MS analysis revealed that the stock solution was impure and contained 80% 9-oxo-ODE, 11% 5,6-DiHETrE and 9% EPA. Regardless, no significant changes in somatic or dendritic PPF were observed with 9-oxo-ODE.

The purity of the stock LA and AA was determined by gas-chromatography (GC), whereas that of 13-HODE, PGE_2_ and 14,15-EET was measured by LC-MS/MS. An 1 ml aliquot of ACSF containing the metabolites was obtained at the end of the 10-minute perfusion to determine whether the compounds were modified by being maintained at 37 °C in oxygenated ACSF (95% O_2_−5% CO_2_ for 10 minutes). The purity of the compounds was measured on an Agilent 1290 Infinity UHPLC system coupled to a 6460 triple-quadrupole tandem mass spectrometer with electrospray ionization (Agilent Corporation, Palo Alto, CA, USA). The system used optimized multiple reaction monitoring (MRM) conditions and was operated in negative electrospray ionization mode. Oxylipins were separated on an Agilent Eclipse Plus C-18 reverse-phase column (2.1 × 150 mm, 1.8 µm particle size). The auto-sampler temperature was kept at 4 °C and the column at 45 °C. Mobile phase A contained ultrapure water with 0.1% acetic acid. Mobile phase B contained acetonitrile/methanol (80/15 v/v) with 0.1% acetic acid. The flow rate started at 0.3 mL/min, decreased to 0.2 mL/min between 6 and 6.1 min, held at 0.2 mL/min for 24.4 min, increased to 0.35 mL/min between 30.5 and 30.6 min, held at 0.35 mL/min for 2.1 min, and decreased to 0.3 mL/min between 32.7 and 34 min. The following elution gradient was applied: mobile phase B was held at 40% for 6.1 min, increased to 80% from 6.1 to 20 min, increased to 82% from 20 to 30 min, increased to 99% from 30.5 to 30.6 min, held at 99% for 2 min, decreased to 40% between 32.6 and 32.7 min and held at 40% for 1.3 min. Peaks were analyzed using Agilent Mass Hunter Workstation Software Quantitative Analysis for QQQ (Version B.07.00). The oxylipin panel assayed included 72 available compounds from Cayman Chemicals and Larodan. The panel included all compounds listed in Supplementary Table 1, less the following: THF diols, EKODE, 15(16)-EpODE, 12(13)-EpODE, 9(10)-EpODE, 15,16-DiHODE, 12,13-DiHODE, 9,10-DiHODE, 19,20-DiHDPE, 16,17-DiHDPE, 13,14-DiHDPE, 10,11-DiHDPE, 7,8-DiHDPE, 4,5-DiHDPE, 11,12-DiHETE, 8,9-DiHETE, 11,12,15-TriHETrE and LTB5. It also included leukotrienes C4, D4 and E4.

### Statistical analysis

Data were expressed as mean ± standard deviation (SD). Oxylipin extraction and analysis, as well as the analysis of extracellular recordings were performed by blinded individuals.

Differences between the CO_2_-group and MW-fixed controls were assessed using an unpaired t-test (GraphPad Prism 6.0, GraphPad Software Inc., San Diego, CA, USA). The final sample size per group was between 7 and 9, because the surrogate standard peak could not be accurately integrated for some of the samples. Heat maps were generated using MetaboAnalyst 3.0^94, 95^.

Somatic and dendritic PPF were expressed relative to the average baseline per slice. Absolute PPF data are presented in Supplementary Table 2. The effect of time and test compound on the normalized somatic and dendridic PPF were evaluated with a two-way repeated measures ANOVA (GraphPad Prism 6.0, GraphPad Software Inc., San Diego, CA, USA). When a significant interaction was found, Dunnett’s multiple comparison test was performed to evaluate for each time point the effect of the test compound compared to vehicle. The analysis was performed on the minute-by-minute data, as well as on the average data per period (baseline – compound – washout).

Statistical significance was set at *p* < 0.05.

## Electronic supplementary material

Supplementary information
