# Supplementary material for: Linoleic acid participates in the response to ischemic brain injury through oxidized metabolites that regulate neurotransmission
Source: Sci Rep. 2017 Jun 28;7:4342. doi: 10.1038/s41598-017-02914-7 (PMC5489485; doi:10.1038/s41598-017-02914-7)

**Linoleic acid participates in the response to ischemic brain injury through oxidized metabolites that regulate neurotransmission**

Marie Hennebelle^1^, Zhichao Zhang^1^, Adam H. Metherel^2^, Alex P. Kitson^2^, Yurika Otoki^1,3^, Christine E. Richardson^4^, Jun Yang^5^, Kin Sing Stephen Lee^5^, Bruce D. Hammock^5^, Liang Zhang^6,7^, Richard P. Bazinet^2^ and Ameer Y. Taha*^1^

^1^Department of Food Science and Technology, College of Agriculture and Environmental Sciences, University of California, Davis, CA, USA; ^2^Department of Nutritional Sciences, Faculty of Medicine, University of Toronto, ON, Canada; ^3^Food and Biodynamic Laboratory, Graduate School of Agricultural Science, Tohoku University, Sendai, Miyagi, Japan; ^4^Department of Nutrition, College of Agriculture and Environmental Sciences, University of California, Davis, CA, USA; ^5^Department of Entomology and Nematology, College of Agriculture and Environmental Sciences and Comprehensive Cancer Center, Medical Center, University of California, Davis, CA, USA; ^6^Krembil Research Institute, University Health Network, Toronto, ON, Canada and ^7^Department of Medicine (Neurology), University of Toronto, ON, Canada

*Corresponding author:

Dr. Ameer Y. Taha

Department of Food Science and Technology, College of Agriculture and Environmental Sciences, University of California Davis, One Shields Avenue, Davis, CA, USA 95616

Phone: (+1) 530-752-7096; Email: [ataha@ucdavis.edu](mailto:ataha@ucdavis.edu)

**Supplementary Table 1.** List of oxylipins measured by LC-MS/MS

|  | **Abbreviation** | **Precursor ion *m/z*** | **Parent ion *m/z*** | **Retention time** |
| --- | --- | --- | --- | --- |
| **Linoleic acid (LA, 18:2 n-6) metabolites:** |  |  |  |  |
| - 9-hydroxyoctadecadienoic acid | 9-HODE | 295.2 | 171.1 | 12.3 |
| - 13-hydroxyoctadecadienoic acid | 13-HODE | 295.2 | 195.2 | 12.22 |
| - 9-oxo-octadecadienoic acid | 9-oxo-ODE | 293.2 | 185.1 | 13.38 |
| - 13-oxo-octadecadienoic acid | 13-oxo-ODE | 293.2 | 195.1 | 13.16 |
| - 12(13)-epoxyoctadecamonoenoic acid | 12(13)-EpOME | 295.3 | 195.2 | 14.9 |
| - 9(10)-epoxyoctadecamonoenoic acid | 9(10)-EpOME | 295.3 | 171.1 | 15.16 |
| - Tetrahydrofuran-diols | THF diol | 353.2 | 127.1 | 6.77 |
| - Epoxyketooctadecenoic acid | EKODE | 309.2 | 291.1 | 10.45 |
| - 12,13-dihydroxyoctadecamonoenoic acid | 12,13-DiHOME | 313.2 | 183.2 | 8.45 |
| - 9,10-dihydroxyoctadecamonoenoic acid | 9,10-DiHOME | 313.2 | 201.2 | 8.82 |
| - 9,12,13-trihydroxyoctadecamonoenoic acid | 9,12,13-TriHOME | 329.2 | 211.1 | 4.49 |
| - 9,10,13-trihydroxyoctadecamonoenoic acid | 9,10,13-TriHOME | 329.2 | 171.1 | 4.57 |
| **Di-homo-gamma-linolenic acid (DGLA, 20:3 n-6) metabolites:** |  |  |  |  |
| - 15(S)-hydroxyeicosatrienoic acid | 15(S)-HETrE | 321.2 | 221.2 | 14.17 |
| - Prostaglandin E1 | PGE1 | 353.3 | 317.2 | 4.85 |
| - Prostaglandin D1 | PGD1 | 353.3 | 317.2 | 4.97 |
| **Arachidonic acid (AA, 20:4 n-6) metabolites** |  |  |  |  |
| - 14(15)-epoxyeicosatrienoic acid | 14(15)-EpETrE | 319.2 | 219.3 | 15.1 |
| - 8(9)-epoxyeicosatrienoic acid | 8(9)-EpETrE | 319.2 | 155.2 | 15.98 |
| - 11(12)-epoxyeicosatrienoic acid | 11(12)-EpETrE | 319.3 | 167.2 | 15.76 |
| - 5(6)-epoxyeicosatrienoic acid | 5(6)-EpETrE | 319.2 | 191.1 | 16.22 |
| - 20-hydroxyeicosatetraenoic acid | 20-HETE | 319.2 | 275.1 | 11 |
| - 6-trans-leukotriene B4 | 6-trans-LTB4 | 335.2 | 195.1 | 7.61 |
| - Leukotriene B4 | LTB4 | 335.2 | 195.1 | 7.88 |
| - 20-OH-Leukotriene B4 | 20-OH-LTB4 | 351.2 | 195.2 | 3.58 |
| - 20-COOH- Leukotriene B4 | 20-COOH-LTB4 | 365.2 | 347.2 | 3.4 |
| - Tromboxane B2 | TXB2 | 369.2 | 169.1 | 4.13 |
| - 6-keto-prostaglandin F1-α | 6-keto-PGF1α | 369.3 | 163.2 | 3.45 |
| - Prostaglandin F2-α | PGF2α | 353.2 | 309.2 | 4.54 |
| - Prostaglandin E2 | PGE2 | 351.2 | 271.3 | 4.72 |
| - Prostaglandin D2 | PGD2 | 351.2 | 271.3 | 4.99 |
| - Prostaglandin J2 | PGJ2 | 333.3 | 189.2 | 6.53 |
| - Prostaglandin B2 | PGB2 | 333.3 | 175.1 | 6.66 |
| - 15-deoxy-Prostaglandin J2 | 15-deoxy-PGJ2 | 315.2 | 271.2 | 10.86 |
| - 5-hydroxyeicosatetraenoic acid | 5-HETE | 319.2 | 115.2 | 14.44 |
| - 15-hydroxyeicosatetraenoic acid | 15-HETE | 319.2 | 219.2 | 12.46 |
| - 11-hydroxyeicosatetraenoic acid | 11-HETE | 319.2 | 167.2 | 13.2 |
| - 8-hydroxyeicosatetraenoic acid | 8-HETE | 319.2 | 155.2 | 13.57 |
| - 12-hydroxyeicosatetraenoic acid | 12-HETE | 319.2 | 179.2 | 13.57 |
| - 9-hydroxyeicosatetraenoic acid | 9-HETE | 319.2 | 167.2 | 13.7 |
| - 15-oxo-eicosatetraenoic acid | 15-oxo-ETE | 317.2 | 113.1 | 13.64 |
| - 12-oxo-eicosatetraenoic acid | 12-oxo-ETE | 317.2 | 153.1 | 14.07 |
| - 5-oxo-eicosatetraenoic acid | 5-oxo-ETE | 317.2 | 273.2 | 15.51 |
| - Lipoxin A4 | LXA4 | 351.2 | 115.2 | 5.33 |
| - 14,15-dihydroxyeicosatrienoic acid | 14,15-DiHETrE | 337.2 | 207.1 | 9.29 |
| - 11,12-dihydroxyeicosatrienoic acid | 11,12-DiHETrE | 337.2 | 167.1 | 10.15 |
| - 8,9-dihydroxyeicosatrienoic acid | 8,9-DiHETrE | 337.2 | 127.1 | 10.65 |
| - 5,6-dihydroxyeicosatrienoic acid | 5,6-DiHETrE | 337.2 | 145.1 | 11.06 |
| - 11,12,15-trihydroxyeicosatrienoic acid | 11,12-,15-TriHETrE | 353.2 | 167.1 | 5.36 |
| - 8,15-dihydroxyeicosatetraenoic acid | 8,15-DiHETE | 335.2 | 235.2 | 7.26 |
| - 5,15-dihydroxyeicosatetraenoic acid | 5,15-DiHETE | 335.3 | 173.2 | 7.57 |
| **α-linolenic acid (α-LNA, 18:3 n-3) metabolites:** |  |  |  |  |
| - 9- hydroxyoctadecatrienoic acid acid | 9-HOTrE | 293.2 | 171.2 | 10.19 |
| - 13- hydroxyoctadecatrienoic acid acid | 13-HOTrE | 293.2 | 195.1 | 10.45 |
| - 15(16)-epoxyoctadecadienoic acid | 15(16)-EpODE | 293.3 | 235.2 | 12.65 |
| - 12(13)-epoxyoctadecadienoic acid | 12(13)-EpODE | 293.2 | 183.1 | 13.32 |
| - 9(10)-epoxyoctadecadienoic acid | 9(10)-EpODE | 293.3 | 171.2 | 12.98 |
| - 15,16-dihydroxyoctadecadienoic acid | 15,16-DiHODE | 311.2 | 223.2 | 7.22 |
| - 12,13-dihydroxyoctadecadienoic acid | 12,13-DiHODE | 311.2 | 183.1 | 7.33 |
| - 9,10-dihydroxyoctadecadienoic acid | 9,10-DiHODE | 311.2 | 201.2 | 7.29 |
| **Eicosapentaenoic acid (EPA, 20:5 n-3) metabolites:** |  |  |  |  |
| - 17(18)-epoxyeicosateteaenoic acid | 17(18)-EpETE | 317.2 | 215.2 | 12.83 |
| - 11(12)-epoxyeicosateteaenoic acid | 11(12)-EpETE | 317.2 | 167.2 | 13.75 |
| - 8(9)-epoxyeicosateteaenoic acid | 8(9)-EpETE | 317.2 | 127.2 | 13.99 |
| - 15-hydroxyeicosapentaenoic acid | 15-HEPE | 317.2 | 219.2 | 10.89 |
| - 12-hydroxyeicosapentaenoic acid | 12-HEPE | 317.2 | 179.2 | 13.42 |
| - 8-hydroxyeicosapentaenoic acid | 8-HEPE | 317.2 | 155.2 | 13.58 |
| - 5-hydroxyeicosapentaenoic acid | 5-HEPE | 317.2 | 115.1 | 11.77 |
| - Leukotriene B5 | LTB5 | 333.3 | 195.2 | 6.59 |
| - Prostaglandin E3 | PGE3 | 349.3 | 269.2 | 4.16 |
| - Prostaglandin D3 | PGD3 | 349.3 | 269.2 | 4.38 |
| - Resolvin E1 | Resolvin E1 | 349.3 | 195 | 3.48 |
| - 17,18-dihydroxyeicosatetraenoic acid | 17,18-DiHETE | 335.3 | 247.2 | 7.64 |
| - 14,15-dihydroxyeicosatetraenoic acid | 14,15-DiHETE | 335.3 | 207.2 | 8.12 |
| - 11,12-dihydroxyeicosatetraenoic acid | 11,12-DiHETE | 335.2 | 167.1 | 8.29 |
| - 8,9-dihydroxyeicosatetraenoic acid | 8,9-DiHETE | 335.2 | 127.1 | 8.6 |
| - 5,6-dihydroxyeicosatetraenoic acid | 5,6-DiHETE | 335.2 | 115.2 | 10.71 |
| **Docosahexaenoic acid (DHA, 22:6 n-3) metabolites:** |  |  |  |  |
| - 19(20)-epoxydocosapentaenoic acid | 19(20)-EpDPE | 343.2 | 241.2 | 14.84 |
| - 16(17)-epoxydocosapentaenoic acid | 16(17)-EpDPE | 343.2 | 233.2 | 15.37 |
| - 13(14)-epoxydocosapentaenoic acid | 13(14)-EpDPE | 343.2 | 193.2 | 15.49 |
| - 10(11)-epoxydocosapentaenoic acid | 10(11)-EpDPE | 343.2 | 153.2 | 15.63 |
| - 7(8)-epoxydocosapentaenoic acid | 7(8)-EpDPE | 343.2 | 113.1 | 15.89 |
| - 17- hydroxydocosahexaenoic acid | 17-HDoHE | 343.2 | 281.2 | 13.12 |
| - 19,20-dihydroxydocosapentaenoic acid | 19,20-DiHDPE | 361.2 | 273.2 | 9.48 |
| - 16,17-dihydroxydocosapentaenoic acid | 16,17-DiHDPE | 361.2 | 233.2 | 9.66 |
| - 13,14-dihydroxydocosapentaenoic acid | 13,14-DiHDPE | 361.2 | 193.2 | 10.23 |
| - 10,11-dihydroxydocosapentaenoic acid | 10,11-DiHDPE | 361.2 | 153.2 | 10.5 |
| - 7,8-dihydroxydocosapentaenoic acid | 7,8-DiHDPE | 361.2 | 113.1 | 10.82 |
| - 4,5-dihydroxydocosapentaenoic acid | 4,5-DiHDPE | 361.2 | 229.3 | 12.14 |

**Supplementary Table 2.** Paired-pulse facilitation (PPF) ratio for somatic and dendritic responses recorded in the CA1 region of the hippocampus. The slices were perfused with vehicle, 1 µM linoleic acid (LA), 1 µM arachidonic acid (AA), 0.1 µM or 1 µM 13-hydroxyoctadecadienoic acid (13-HODE), or 0.1 µM or 1 µM Prostaglandin E2 (PGE_2_). Data are mean ± SD of n= 4-6 slices per condition. Data were analyzed by a 2-way repeated measures ANOVA followed by Dunnett's multiple comparison test. Asterisks (*) indicates significant differences for each recording period (baseline, compound or washout) compared to vehicle. Different letter superscripts (a,b) reflect significant differences amongst the different recording periods per treatment (vehicle, LA, AA, 13-HODE or PGE2).

|  |  | **Baseline** | **Compound** | **Washout** |
| --- | --- | --- | --- | --- |
| **Average somatic PPF** | **Vehicle** | 2.4 ± 1.4 | 2.5 ± 1.5 | 2.6 ± 1.2 |
|  | **1 µM LA** | 3.1 ± 2.5 | 3.2 ± 2.7 | 3.4 ± 2.5 |
|  | **1 µM AA** | 2.0 ± 0.4 | 2.0 ± 0.6 | 2.6 ±1.0 |
|  | **0.1 µM 13-HODE** | 3.1 ± 2.1^a^ | 4.2 ± 3.8^a^ | 6.4 ±6.7^b^* |
|  | **1 µM 13-HODE** | 2.4 ± 0.6 | 2.5 ± 1.2 | 3.3 ± 2.7 |
|  | **0.1 µM PGE2** | 2.5 ± 1.7^a^ | 3.7 ± 3.9^a^ | 8.2 ± 9.5^b^* |
|  | **1 µM PGE2** | 1.7 ± 0.3 | 1.5 ± 0.2 | 1.5 ± 0.2 |
| **Average dendritic PPF** | **Vehicle** | 1.7 ± 0.3 | 1.8 ±0.3 | 1.8 ± 0.2 |
|  | **1 µM LA** | 1.4 ± 0.2 | 1.5 ±0.2 | 1.5 ± 0.3 |
|  | **1 µM AA** | 1.5 ± 0.3 | 1.5 ±0.2 | 1.5 ± 0.3 |
|  | **0.1 µM 13-HODE** | 1.8 ± 0.5 | 1.8 ± 0.5 | 1.9 ± 0.4 |
|  | **1 µM 13-HODE** | 1.6 ± 0.2 | 1.6 ±0.2 | 1.7 ± 0.3 |
|  | **0.1 µM PGE2** | 1.8 ± 0.4 | 1.8 ±0.5 | 1.8 ± 0.5 |
|  | **1 µM PGE2** | 1.5 ± 0.2 | 1.5 ± 0.2 | 1.5 ± 0.2 |

**Supplementary Figure 1.** Representative recordings of somatic and dendritic responses following paired-pulse stimulation.


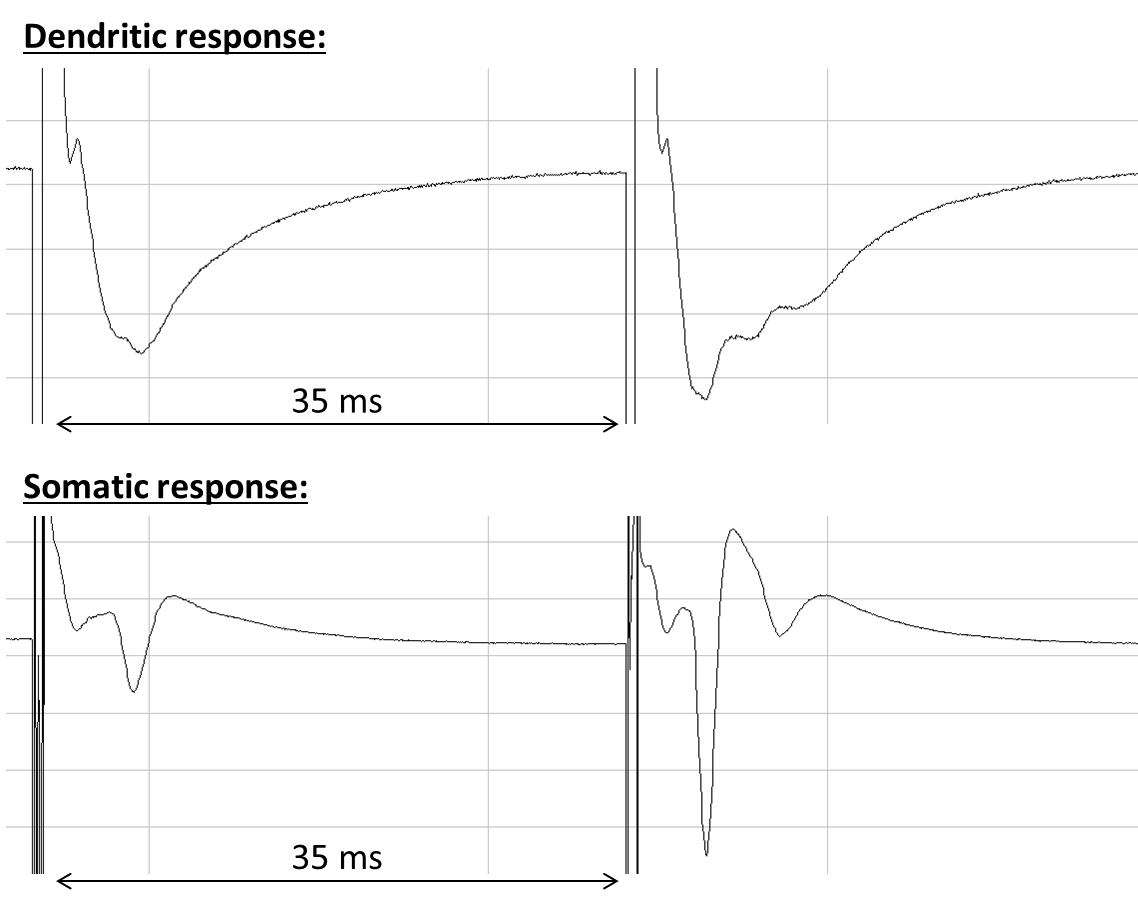


**Supplementary Figure 2.** Somatic (a. and b.) and dendritic (c. and d.) paired-pulse facilitation (PPF) of hippocampal evoked responses following perfusion with vehicle or 1 µM 14,15-epoxyeicosatrienoic acid (14,15-EET). Data (mean ± SD) are expressed relative to baseline (n=4-6 per condition). Graphs a. and c. show the minute-by-minute data; b. and d. show average PPF during compound incubation and washout relative to baseline (dotted line). Asterisks (*) indicates significant differences compared to vehicle as determined by a two-way repeated measures ANOVA followed by Dunnett’s multiple comparison test.


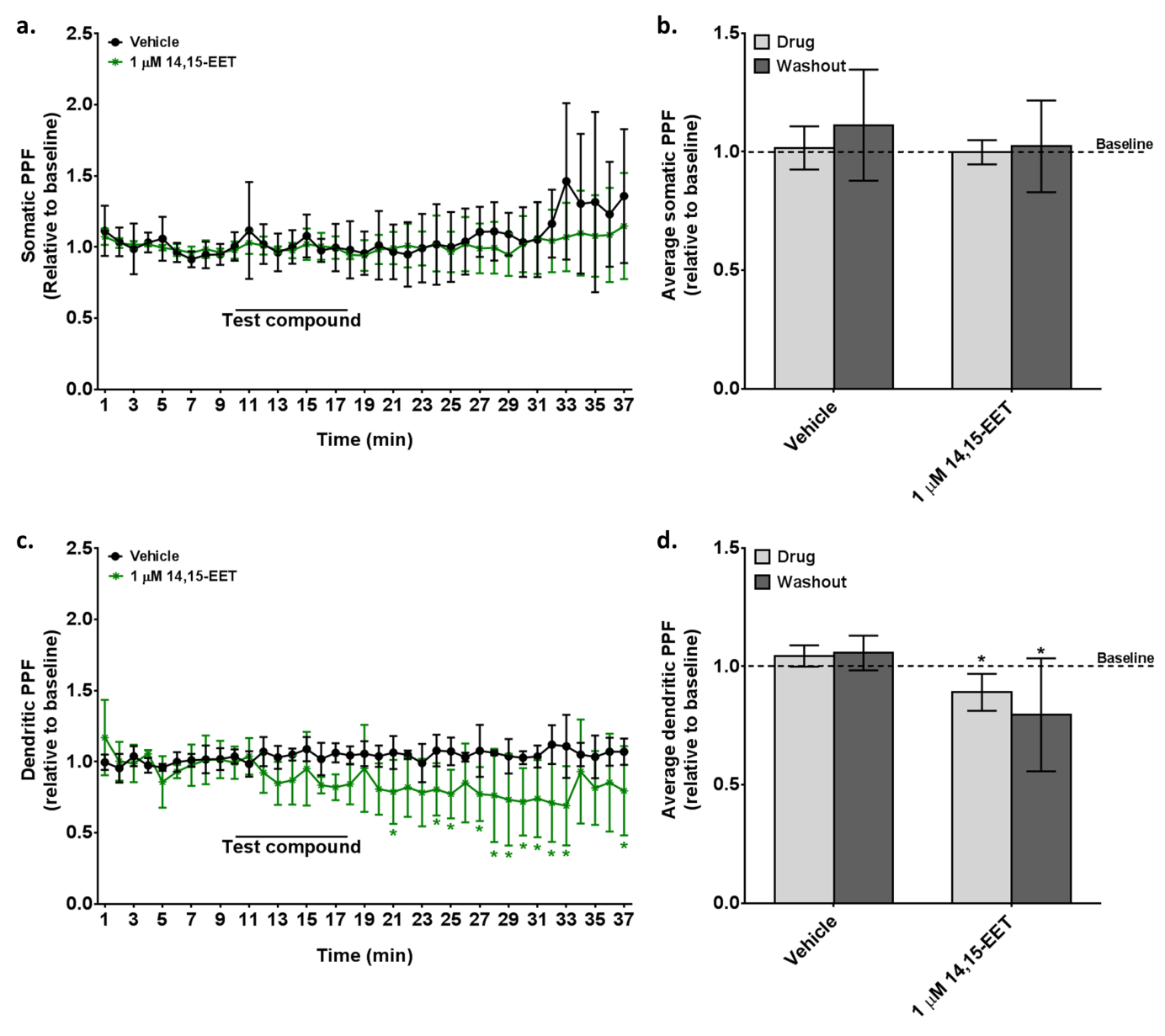

Supplement: Supplementary file 1 — Supplementary information [file 41598_2017_2914_MOESM1_ESM.docx]
